# Supplementary material for: Functional Characterization of Heat Shock Factor (CrHsf) Families Provide Comprehensive Insight into the Adaptive Mechanisms of Canavalia rosea (Sw.) DC. to Tropical Coral Islands
Source: Int J Mol Sci. 2022 Oct 15;23(20):12357. doi: 10.3390/ijms232012357 (PMC9604225; doi:10.3390/ijms232012357)
Supplement: Supplementary file 1 [file ijms-23-12357-s001.zip › Supplementary material of CrHsfs Ms-20221014.pdf]

Supplementary Material Information

Functional characterization of heat shock factors (*CrHsf*s) family provides comprehensive insight into the adaptive mechanisms of *Canavalia rosea* (Sw.) DC. to tropical coral islands

Mei Zhang [1,2,*], Zhengfeng Wang [1,3,4] and Shuguang Jian [1,3,*]

[1] Guangdong Provincial Key Laboratory of Applied Botany, South China Botanical Garden, Chinese Academy of Sciences, Guangzhou 510650, China; zhangmei@scbg.ac.cn (M.Z.); wzf@scbg.ac.cn (Z.W.); jiansg@scbg.ac.cn (S.J.)
[2] Key Laboratory of South China Agricultural Plant Molecular Analysis and Genetic Improvement, South China Botanical Garden, Chinese Academy of Sciences, Guangzhou 510650, China
[3] Key Laboratory of Vegetation Restoration and Management of Degraded Ecosystems, South China Botanical Garden, Chinese Academy of Sciences, Guangzhou 510650, China
[4] Key Laboratory of Carbon Sequestration in Terrestrial Ecosystem, South China Botanical Garden, Chinese Academy of Sciences, Guangzhou 510650, China
* Correspondence: zhangmei@scbg.ac.cn (M.Z.) and jiansg@scbg.ac.cn (S.J.)

Figure S1. Potential interactions among the CrHsf proteins based on the soybean database predicted by the STRING database (https://string-db.org/).

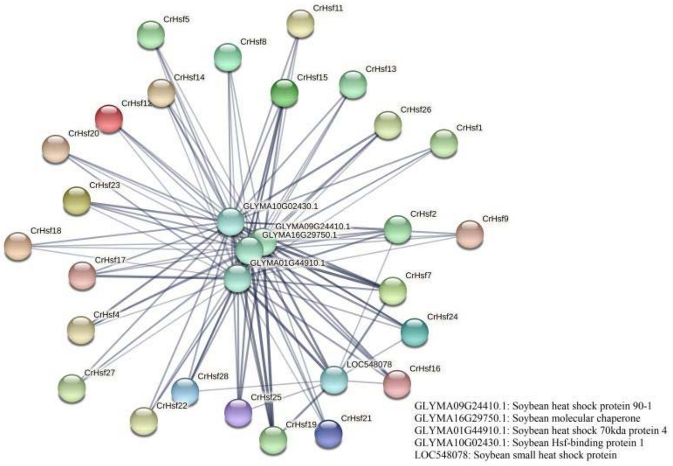

Figure S2. The high salinity and high osmotic stress tolerance confirmation of wild type (WT) yeast mediated by eight *CrHsf*s on solid medium containing NaCl (A) or sorbitol (B). The WT strain BY4741 was transformed with the empty vector pYES2, and the mutant strains were transformed with pYES2 or with recombinant vectors *CrHsf*s-pYES2. Yeast cultures were adjusted to OD600 =2, and 2 μL of serial dilutions (10 fold, from left to right in each panel) were spotted on SDG/-Ura medium supplemented with different concentrations of NaCl or sorbitol. The corresponding yeast spots growing on SDG/-Ura plates without any stress factor were used as controls. The plates were incubated for 2–5 days at 30°C.

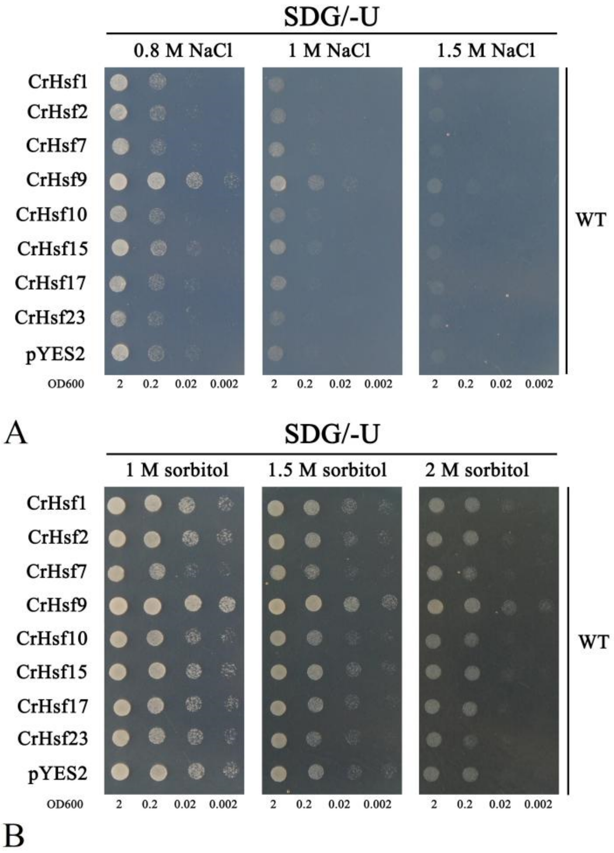

Table S1. The sequences of *CrHsf* genomic DNA, CDS, and promoter region DNA.

Table S2. Summary of possible candidate *cis*-regulatory elements found in *CrHsf* promoter DNA regions.

Table S3. The FPKM values of *CrHsf*s for RNA-Seq assay of *C. rosea* tissues in this study.

Table S4. Primer sequences used in this study.
